# Metal organic framework synthesis in the presence of surfactants: towards hierarchical MOFs?†
†Electronic supplementary information (ESI) available. See DOI: 10.1039/c4ce02324b



**DOI:** 10.1039/c4ce02324b

**Published:** 2015-01-23

**Authors:** B. Seoane, A. Dikhtiarenko, A. Mayoral, C. Tellez, J. Coronas, F. Kapteijn, J. Gascon

**Affiliations:** a Catalysis Engineering , ChemE , Delft University of Technology , Julianalaan 136 , 2628 BL Delft , The Netherlands . Email: B.SeoanedelaCuesta@tudelft.nl ; Email: j.gascon@tudelft.nl ; Fax: +31 1527 85006 ; Tel: +31 1527 84851; b Chemical and Environmental Engineering Department and Nanoscience Institute of Aragon (INA) , Universidad de Zaragoza , Mariano Esquillor , Edificio I+D , 50018 , Zaragoza , Spain; c Advanced Microscopy Laboratory (LMA) , Nanoscience Institute of Aragon (INA) , Universidad de Zaragoza , Mariano Esquillor , Edificio I+D , 50018 , Zaragoza , Spain

## Abstract

The effect of synthesis pH and H_2_O/EtOH molar ratio on the textural properties of different aluminium trimesate metal organic frameworks (MOFs) prepared in the presence of the well-known cationic surfactant cetyltrimethylammonium bromide (CTAB) at 120 °C was studied with the purpose of obtaining a MOF with hierarchical pore structure. Depending on the pH and the solvent used, different topologies were obtained (namely, MIL-96, MIL-100 and MIL-110). On the one hand, MIL-110 was obtained at lower temperatures than those commonly reported in the literature and without additives to control the pH; on the other hand, MIL-100 with crystallite sizes as small as 30 ± 10 nm could be easily synthesized in a mixture of H_2_O and EtOH with a H_2_O/EtOH molar ratio of 3.4 at pH 2.6 in the presence of CTAB. The resulting material displays a hierarchical porosity that combines the microporosity from the MOF and the non-ordered mesopores defined in between the MOF nanoparticles. Interestingly, the maximum of the pore size distribution could be varied between 3 and 33 nm. Finally, at pH 2.5 and using water as a solvent, platelets of MIL-96, a morphology never observed before for this MOF, were synthesized with a (001) preferential crystal orientation, the (001) plane running parallel to the bipyramidal cages of the MIL-96 topology.

## Abstract 2

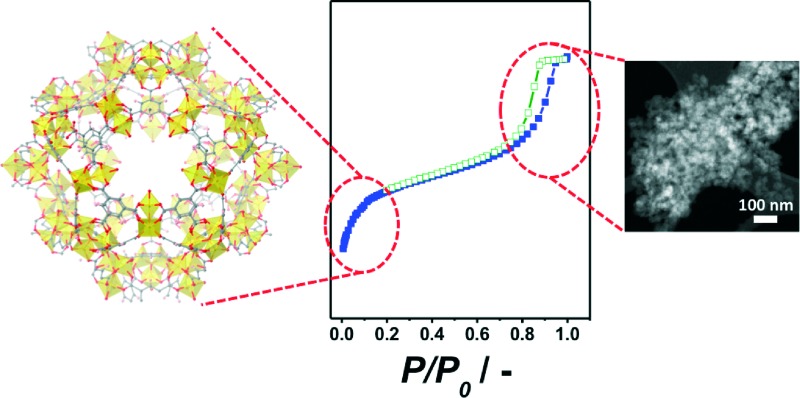
We report the effect of synthesis parameters on the textural properties of Al based MOFs synthesized in the presence CTAB.

## A Introduction

Metal–organic frameworks (MOFs) are ordered porous crystalline materials resulting from the self-assembly of metal ions or clusters with organic linkers possessing carboxylates, phosphonates, sulfonates or N-containing multidentate ligands.^1^ Due to their outstanding textural properties,^2^ flexibility^3^ and rich pre-^4,5^ and post-^6^ synthesis chemistry, MOFs are very versatile architectures with promising applications in the fields of adsorption,^2^ encapsulation,^7^ drug delivery,^8^ catalysis,^9^ membranes^10,11^ and separation and storage of gases and vapors,^11^ among others. However, while high micropore volumes and large surface areas are desirable for many applications, such narrow pores do not allow inclusion or anchoring of bulky host molecules. Moreover, diffusive transport in micropores may limit catalytic and separation performance;^12^ therefore, the synthesis of MOFs with a hierarchical pore structure, combining pores below and over 2 nm, would offer several advantages.

In this spirit, different approaches have been reported to manufacture mesoporous MOFs (2 nm < *d*_p_ < 50 nm).^13–15^ As template free synthesis strategies, ligand extension, microemulsion methods and nanocrystal self-assembly have been reported. Furthermore, different surfactants have been used to obtain mesoporous MOFs where a long-range order has been claimed.

Ligand extension, or more precisely SBU extension, is an attractive strategy leading to the synthesis of different MOFs with mesoporous channels such as IRMOF-16^4^ and MesoMOF-1^16^ or cavities like MIL-100,^17^ MIL-101,^18,19^ ZIF-95, ZIF-100^20^ and NU-100 (also termed PCN-610).^21^ However, introducing longer bridging ligands presents difficulties.^22^ On the one hand, the product obtained often exhibits framework interpenetration to maximize the packing efficiency;^23^ on the other hand, the structure may collapse upon guest removal. Together with SBU extension, other surfactant-free approaches such as microemulsions and nanocrystal assembly have also been applied to obtain MOFs with mesopores. As an example of the latter, Yue *et al.* ^24^ reported the synthesis of Zn–MOF-74 with disordered mesopores with widths of 5–20 nm formed between the nanosized MOF crystals.

The use of surfactants as structure directing agents has been extensively applied to the supramolecular template-directed synthesis of different materials such as mesoporous silicas, aluminosilicates and other mesostructured metal oxides. Thus, it also seems to be an attractive strategy to obtain mesoporosity in MOFs.^25^ Since the pioneering work of Roy *et al.*,^26,27^ in which the synthesis of the first liquid-crystal templated mesoporous MOF was accomplished with self-made surfactants that bind alkyl chains to the organic ligands of different Prussian blue analogues, several examples of surfactant templated MOFs have been published. Qui *et al.* ^28^ reported the synthesis of HKUST-1 in the presence of the cationic surfactant cetyltrimethylammonium bromide (CTAB) and the organic compound 1,3,5-trimethylbenzene (TMB) as a micelle swelling agent. In contrast to other mesoporous materials such as silicas that exhibit amorphous walls, Qui *et al.* obtained a hierarchical MOF in which the walls of the mesopores were composed of crystalline microporous HKUST-1.^28^ Zhao *et al.* ^29^ synthesized nanospheres with long-range ordered mesopores in an emulsion system containing an ionic liquid, supercritical CO_2_ and the surfactant IL/SCCO_2_/N-EtFOSA and Ma *et al.* ^30^ reported the synthesis of crystalline metal disulfonates with well-structured hexagonal mesoporosity controlling the release of the metal ions by a crown ether and using F-127 as a non-ionic surfactant.

One of the most studied subclasses of MOFs is the MIL (MIL stands for Material Institute Lavoisier) family in which the metal node is commonly a trivalent cation and the ligand a di-, tri- or tetracarboxylic acid. Employing 1,3,5-benzenetricarboxylic acid (trimesic acid) as a linker and aluminium as the metal node, three different topologies have been reported: MIL-96,^31^ MIL-100^32^ and MIL-110.^33^ In all these structures the aluminium atoms are octahedrally coordinated. However, while MIL-110 exhibits inorganic octameric motifs connected by trimesate molecules to form hexagonal 16 Å channels,^33^ MIL-96 and MIL-100 contain trimeric units. MIL-100 displays the MTN zeolitic topology with mesoporous cavities of 25 and 29 Å and microporous pentagonal and hexagonal windows of 4.8 × 5.8 Å and 8.6 × 8.6 Å, respectively.^32^ MIL-96 contains isolated trinuclear clusters of μ_3_-O bridged metallic octahedra together with infinite corrugated chains of AlO_4_(OH)_2_ and AlO_2_(OH)_3_(H_2_O), generating a hexagonal network with three types of cavities, two of them having a trigonal bipyramidal shape and a free diameter of 8.8 Å.^31^

In this manuscript we present a thorough study on the effect of different synthesis conditions on the textural properties of different aluminium trimesate MOFs when synthesized in the presence of CTAB. This strategy enabled the synthesis of MOFs with hierarchical porosity combining both the micropores of the MOF topology and the mesopores created by the aggregation of the MOF nanoparticles.

## B Experimental

### Synthesis

In a typical synthesis, 0.844 g of Al(NO_3_)_3_·9H_2_O (≥98%, Sigma-Aldrich) was dissolved in 15 mL of distilled water, and 0.331 g of trimesic acid (H_3_BTC, ≥97%, Sigma-Aldrich) and 1.004 g of cetyltrimethylammonium bromide (CTAB, ≥98%, Sigma-Aldrich) were dissolved separately in 15 mL of EtOH (≥99%, Sigma-Aldrich). Subsequently, the aqueous solution was poured into the ethanolic solution and stirred for 30 min at RT. The final CTAB/Al and H_2_O/EtOH molar ratios were 0.6 and 3.4, respectively, and the pH of the synthesis solution was 2.1. The mixture was transferred to a Teflon®-lined stainless steel autoclave and heated at 120 °C for 12 h under static conditions. After cooling down, the product was filtered off and washed with fresh EtOH. Finally, to activate the MOFs, 100 mg were suspended in 100 mL of EtOH and kept under reflux at 60 °C overnight, filtered off and dried overnight at room temperature.

To study the influence of different synthesis parameters on the final product, several synthesis conditions were tested. The pH was modified between 2.1 and 2.7 by adding different amounts of tetramethylammonium hydroxide (TMAOH, 25 wt.%, Sigma-Aldrich); the H_2_O/EtOH ratio was changed in the range 1.5 to ∞ and four different values for the CTAB/Al molar ratio (0, 0.3, 0.6 and 1.2) were employed (see Table S1†). Depending on the synthesis conditions, three different phases, MIL-96, MIL-100, and MIL-110, or mixtures of them were obtained.

### Characterization

Data from N_2_ adsorption were collected at 77 K on a Quantachrome Autosorb-6B setup. Prior to the measurements the powder samples were outgassed overnight at 130 °C (heating rate: 1 °C min^–1^). BET surface areas were determined from the adsorption branches according to the criteria reported by Walton *et al.*, Rouquerol *et al.* and de Lange *et al.* ^34–36^ The external specific surface area and the micropore volume were both calculated by means of the *t*-plot method and the micropore specific surface area was calculated by subtracting *S*_EXT_ from *S*_BET_. Finally, pore size distributions were calculated using the Barrett–Joyner–Halenda (BJH) model using the adsorption branches to avoid the influence of N_2_ cavitation (*vide infra*).

XRD was performed at room temperature using a Bruker-AXS D5005 with CoKα radiation (*λ* = 1.7890 Å). All data were collected at room temperature over the angular 2*θ* range 5–40° with a step of 0.011°. In order to confirm the preferred orientation of the laminar MIL-96(Al) crystals, pattern matching (profile refinement) was performed in the range 5–40° using FullProf software.^37^ A Pearson VII function was chosen to generate the line shape of the diffraction peaks. Zero offset, the scale factor, six background terms, profile parameters, preferred orientation obtained using the March–Dollase function and unit cell parameters were refined.

SEM images were acquired with an Inspect F scanning electron microscope (FEI) operating at 10 kV.

Prior to the STEM analyses, the samples were dispersed in EtOH. After sonication a few drops of the suspension were placed onto a holey carbon copper microgrid. STEM analysis was performed using an aberration (*C*_s_) corrected FEI Titan operated at 300 kV, equipped with a Gatan bottom-entry CCD 2 K × 2 K digital camera, an EDS detector for chemical analysis, a STEM (BF/ADF/HAADF detector) module and a CEOS corrector for the electron probe.

Infrared spectra were recorded on a Bruker model IFS66 spectrometer in DRIFT mode in a high temperature cell with CaF_2_ windows. The spectra were collected after accumulation of 128 scans with a resolution of 4 cm^–1^. Before collecting the spectra, the sample was pretreated in the equipment under helium at 393 K for 1 h.

Thermogravimetric analysis was performed in a system provided by Mettler Toledo, model TGA/SDTA851e under air flow of 60 mL min^–1^ at a heating rate of 10 °C min^–1^ up to 850 °C.

## C Results and discussion

**Fig. 1:**
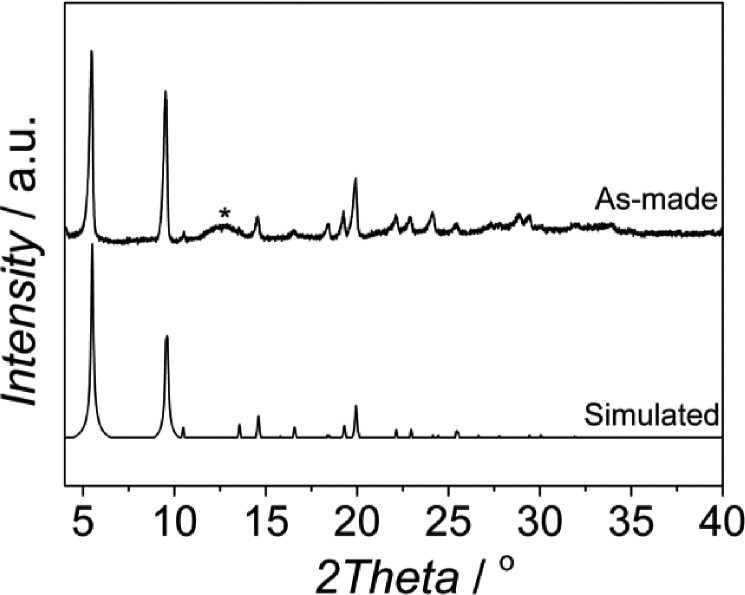
XRD pattern of the sample obtained at 120 °C from a synthesis solution with pH 2.1 and CTAB/Al and H_2_O/EtOH molar ratios of 0.6 and 3.4, respectively, together with the MIL-110 simulated pattern from the data previously reported by Volkringer *et al.* ^33^

Fig. 1 shows the XRD pattern of the sample synthesized from a synthesis solution of pH 2.1 with CTAB/Al and H_2_O/EtOH molar ratios of 0.6 and 3.4, respectively. The reflections observed match with those of the MIL-110 topology^17^ together with some impurities, the latter giving rise to a broad signal at 2*θ* ≈ 12.4°. The presence of impurities in the synthesis of MIL-110 was already reported by Haouas *et al.* ^38^ In fact, similar SEM images were obtained with two different morphologies: needle-like crystals characteristic of MIL-110 and spherical amorphous particles corresponding to the impurities observed by XRD (see Fig. S1†).

In the literature, different methods have been published in order to obtain MIL-110.^33,39–41^ Typically, the synthesis has been carried out in water at 210 °C by controlling the pH using either mineral acid, HNO_3_, or mineral base, NaOH, as a pH adjustment additive.^33,39^ Most of the syntheses reported to date have been performed at pH ≈ 0 or pH ≈ 4, although synthesis at pH 7 has been successful as well.^39,40^ While the most common solvent to obtain MIL-110 is distilled water, the MIL-110 topology has also been observed as an impurity when a mixture of DMF/H_2_O was used.^41^ Besides, the synthesis of MIL-110 aerogels in EtOH at 80 °C has been recently reported. Although the authors claimed that the XRD pattern could not be assigned to a known single MOF phase, the isolated aerogel is, according to the published XRD pattern, related to the MIL-110 network.^42^ In this work, MIL-110 could be obtained using a mixture of water and ethanol at lower temperatures and without additives to control the pH. Furthermore, the BET surface area, calculated from the N_2_ isotherm acquired at 77 K, was 1360 m^2^ g^–1^ which is very close to the value previously reported.^33^ However, even though the synthesis was carried out with a CTAB/Al ratio of 0.6, the isotherm showed a type I behavior and no hysteresis was observed, giving no evidence of any mesoporosity in the sample (see Fig. S2†).

### Influence of pH

The pH was reported to be a parameter with great influence on the synthesis of Al based MOFs with trimesic acid as an organic ligand.^38,43^ Based on these reports, we varied the pH between 2.1 and 2.7. When no base was used (pH 2.1), MIL-110 was formed together with some impurities (Fig. 1).

**Fig. 2:**
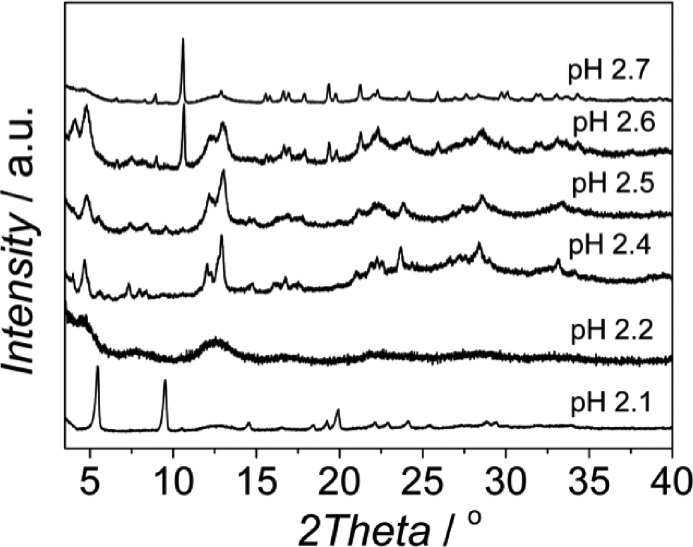
XRD patterns of the samples obtained at different pH with CTAB/Al and H_2_O/EtOH molar ratios of 0.6 and 3.4, respectively.

At higher pH values MIL-100 started to form (see Fig. 2 and Fig. S3†), being the main product in the pH range 2.3–2.5, and at pH 2.6 the sample produced was a mixture of MIL-100 and MIL-96, the relative amount of the latter becoming more important when the pH was further increased. This behavior is different from that previously reported. In water, MIL-110 was isolated at very acidic pH (pH ≈ 0–0.3), MIL-100 was also synthesized in a very narrow pH range (0.5 < pH < 0.7), MIL-96 was obtained at pH 1–3 and MIL-110 appeared again at pH 4.^31,32,39^ Even though the trend could be the same, the pH ranges at which the different topologies were observed are very different under the conditions studied.

**Fig. 3:**
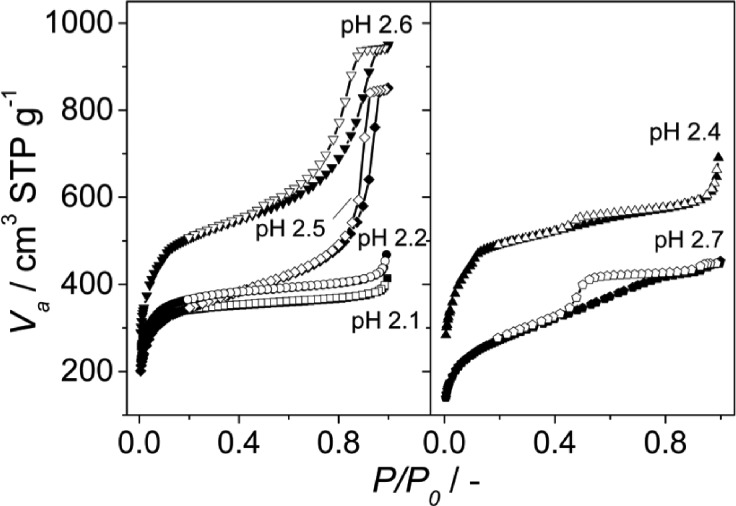
N_2_ adsorption isotherms measured at 77 K on the samples obtained at different pH with CTAB/Al and H_2_O/EtOH molar ratios of 0.6 and 3.4, respectively. The pH values used and the topologies obtained were 2.1 (MIL-110), 2.2 (MIL-100), 2.4 (MIL-100), 2.5 (mixture of MIL-100 and MIL-110) and 2.6 and 2.7 (mixture of MIL-100 and MIL-96); see Table 1. Closed symbols represent adsorption and open symbols desorption branch.

The N_2_ adsorption results and SEM images were in agreement with the XRD patterns obtained. At pH values of 2.1, 2.4 and 2.7, the calculated BET surface areas were 1365 m^2^ g^–1^, 1970 m^2^ g^–1^ and 990 m^2^ g^–1^, respectively (see Fig. 3 and Table 1). The first two values are slightly lower than those previously reported for MIL-110 and MIL-100,^32,33^ suggesting a phase transition upon pH increase. In the case of MIL-96, different data have been published in the literature. Although some publications claimed that the MIL-96 porosity is not accessible to N_2_,^31^ other authors have reported BET surface areas as high as 625 m^2^ g^–1^ together with pore volumes of 0.2 cm^3^ g^–1^.^44^ In this work, the micropore pore volume measured was 0.13 cm^3^ g^–1^. However, the coexistence of MIL-96 with phases with higher porosity such as MIL-100 or MIL-110 must be taken into account.

**Table 1:** Textural properties of the samples obtained at 120 °C and different pH with CTAB/Al and H_2_O/EtOH molar ratios of 0.6 and 3.4, respectively

Run	Phase [MIL]	pH	*S*_BET_ [m^2^ g^–m^]	*S*_INT_ [m^2^ g^–m^]	*S*_EXT_ [m^2^ g^–m^]	*S*_EXT_/*S*_INT_	*V*_MICRO_ [cm^3^ g^–1^]
Al_BTC 1	110	2.1	1360	1170	190	0.16	0.44
Al_BTC 2	100	2.2	1405	1050	360	0.34	0.41
Al_BTC 3	100	2.4	1970	1270	700	0.55	0.49
Al_BTC 4	100/110	2.5	1280	740	530	0.72	0.30
Al_BTC 5	100/96	2.6	1890	1180	720	0.61	0.47
Al_BTC 6	96/100	2.7	990	310	670	2.15	0.13

**Fig. 4:**
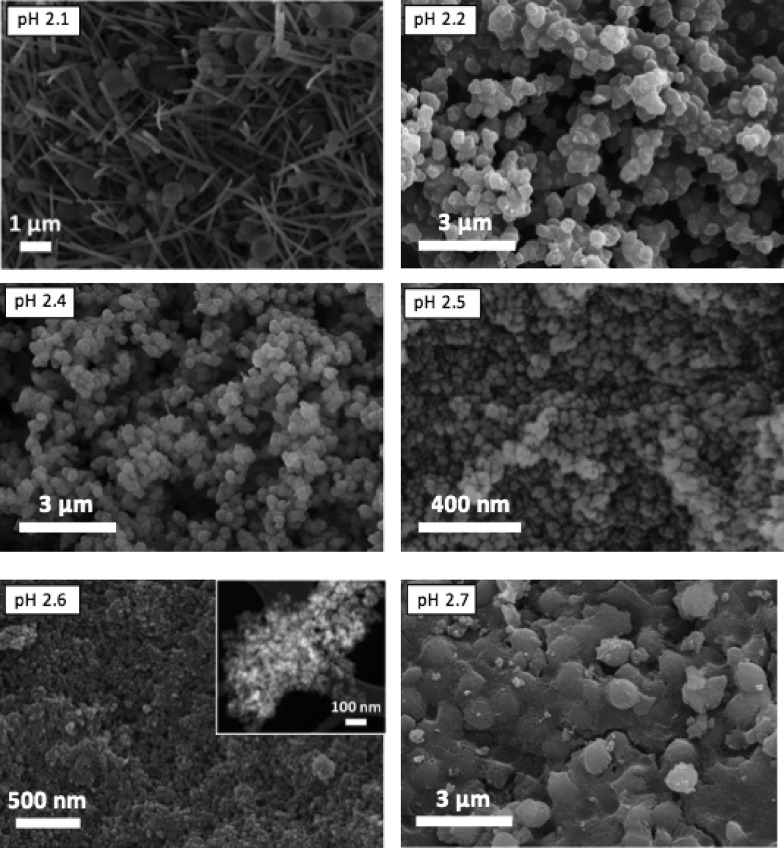
SEM images of the samples obtained at different pH from synthesis solutions with CTAB/Al and H_2_O/EtOH molar ratios of 0.6 and 3.4, respectively. The pH values used and the topologies obtained were 2.1 (MIL-110), 2.2 (MIL-100), 2.4 (MIL-100), 2.5 (mixture of MIL-100 and MIL-110) and 2.6 and 2.7 (mixture of MIL-100 and MIL-96). Inset: TEM image acquired for the MIL-100 sample obtained at pH 2.6.

When the acquired SEM images are considered (see Fig. 4), three different morphologies could be observed, in agreement with the results previously published:^38^ elongated hexagonal crystals characteristic of MIL-110 at pH 2.1, small octahedra in the pH range 2.2–2.5, corresponding to the MIL-100 topology, and a mixture of small octahedra and ill-defined hexagonal crystals due to the coexistence of MIL-100 and MIL-96, respectively, at pH 2.7. The particle size of MIL-100 depends on the pH (Fig. S4†) and decreased from 330 ± 70 nm to sizes as small as 30 ± 5 nm as the amount of TMAOH in the synthesis solution was increased. Indeed, the addition of a base favours the deprotonation of the organic ligand,^45^ increasing its solubility and leading to a more homogenous nucleation, affecting the particle size of the MOF particles and its distribution: the higher the pH (*i.e.* better ligand deprotonation), the more homogeneous and smaller the particle size.

Interestingly, although type I isotherms were obtained for the samples synthesized without TMAOH (pH 2.1), clear hysteresis loops were observed at higher pH. The isotherms of the samples synthesized at pH 2.4 and higher exhibit a behavior between types I and IV with large uptake at low pressures and hysteresis (Fig. 3). The large uptake at low pressures is related to the adsorption in the mesoporous cavities of the MIL-100 (25–30 Å) through its microporous windows (5–8.6 Å), while the hysteresis and slope in the adsorption branch may be attributed to condensation in the interparticle mesoporous voids delimited between the MOF nanocrystals (see Fig. 4, inset).

These results are in agreement with the FTIR and TGA analyses (see Fig. S5 and S6†). At pH 2.1, the C–H stretching band of the CTAB is absent, whereas at pH 2.4 and higher the presence of CTAB in the as-synthesized powder is clearly observed. According to TGA, at pH 2.6 the calculated amount of CTAB in the as-synthesized sample is 6.3 wt.%. We hypothesize that at higher pH, ligand deprotonation is accelerated and the carboxylate moieties, besides coordinating the metal ions, may interact with the cationic surfactant. Interestingly, after treatment with EtOH under reflux and under vacuum at 130 °C, the CTAB was completely removed without affecting the hierarchical porosity of the MOF. The BJH pore size distributions were calculated from the N_2_ isotherms, the sample synthesized at pH 2.5 exhibiting the narrowest pore size distribution with pore diameters around 32 nm (Fig. S7†). It is emphasized that the BJH model was applied to the adsorption branch to avoid the influence of the so-called tensile strength effect (TSE),^46^ indicated by the forced closure of the isotherm at *P*/*P*_0_ = 0.42. When the TSE phenomenon takes place, the BJH model applied to the desorption branch gives a completely different result compared to that obtained from the adsorption branch, where it is absent, leading to the misinterpretation of the pore size distribution. In the former case, a very well defined mesoporosity with a pore size determined primarily by the nature of the adsorptive is obtained. Surprisingly, in recent studies on mesoporous MOFs a very narrow pore size distribution centered at 3.8 nm, caused by the abovementioned TSE effect, was erroneously attributed to the presence of real, very well defined mesoporosity.^42^

Finally, to assess the effect of the surfactant, synthesis at pH 2.5 was also performed without CTAB. The isotherm acquired for the MOF obtained without a surfactant (Fig. S8†) showed no hysteresis but high uptake at high *P*/*P*_0_ (close to *P*/*P*_0_ = 1), pointing to the formation of much bigger MOF crystals and condensation in macropores formed around these bigger particles (see Fig. S9†).

### Influence of EtOH/H_2_O molar ratio

**Fig. 5:**
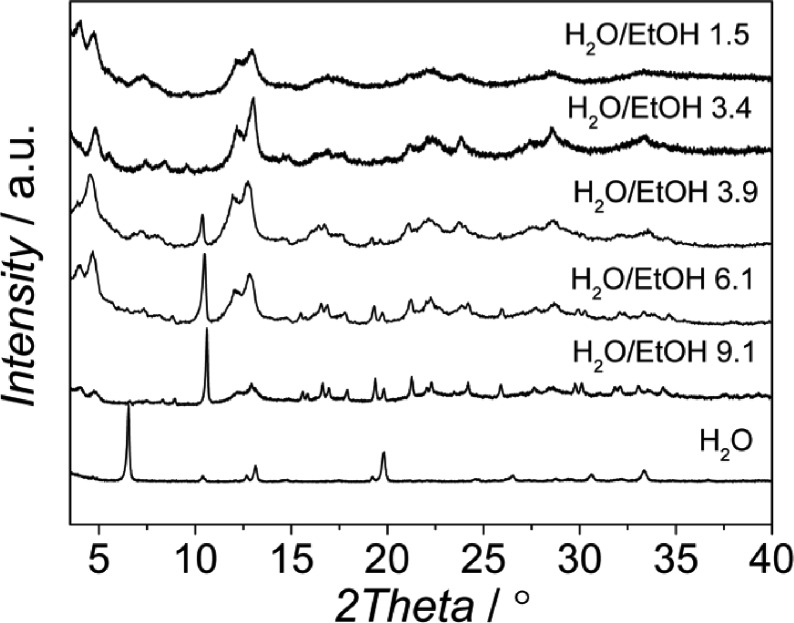
XRD patterns of the samples obtained at pH 2.5 from synthesis solutions with a CTAB/Al molar ratio of 0.6 and different H_2_O/EtOH molar ratios.

The influence of the H_2_O/EtOH molar ratio at pH 2.5 was also investigated (see Table 2). As shown in Fig. 5, mixtures of MIL-96 and MIL-100 were synthesized in the range H_2_O/EtOH 3.9–9.1. As the ratio was decreased, the relative amount of MIL-100 increased. For mixtures with H_2_O/EtOH = 1.5 the powder obtained was already pure MIL-100 and no impurities were observed. This trend suggests that EtOH helps equilibrate the least stable AlBTC (MIL-100), while high concentrations of H_2_O promote the formation of more stable phases (MIL-96)^47^ according to a faster hydrolysis of the kinetic phase at higher water concentrations.^48^

**Table 2:** Textural properties of the samples synthesized at 120 °C and pH 2.5 with a CTAB/Al molar ratio of 0.6 and different H_2_O/EtOH molar ratios

Run	Phase [MIL]	H_2_O/EtOH molar ratio	*S*_BET_ [m^2^ g^–1^]	*S*_INT_ [m^2^ g^–1^]	*S*_EXT_ [m^2^ g^–1^]	*V*_MICRO_ [cm^3^ g^–1^]
Al_BTC 8	96	∞	150	105	50	0.04
Al_BTC 9	96/100	9.1	1180	620	550	0.23
Al_BTC 10	100/96	6.1	1280	720	550	0.28
Al_BTC 11	100/96	3.9	1360	690	670	0.26
Al_BTC 4	100/110	3.4	1280	740	530	0.30
Al_BTC 12	100	1.5	1550	670	880	0.25

**Fig. 6:**
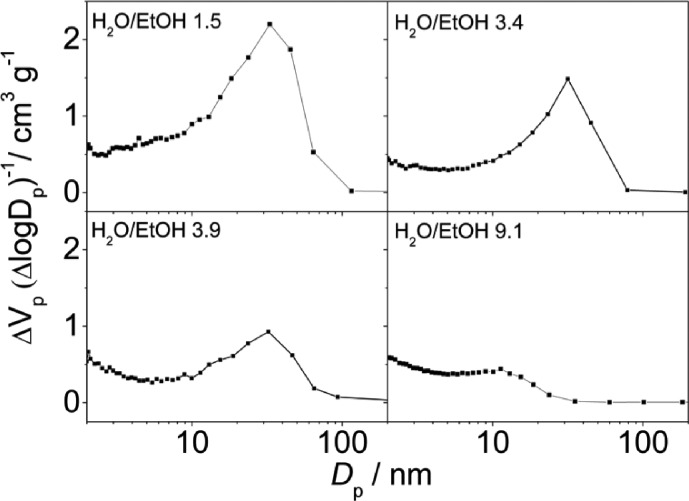
BJH pore size distribution curves of the samples synthesized at pH 2.5 in mixtures of H_2_O and EtOH with different molar ratios and a CTAB/Al ratio of 0.6.

**Fig. 7:**
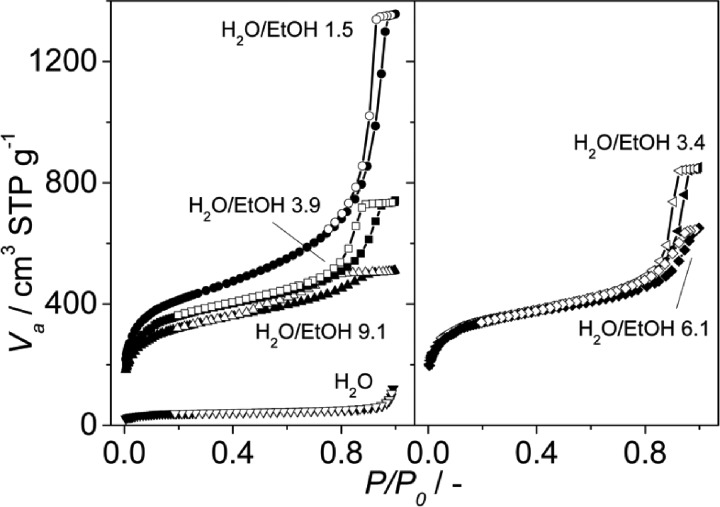
N_2_ adsorption isotherms acquired at 77 K on the samples synthesized at pH 2.5 with different H_2_O/EtOH molar ratios. The H_2_O/EtOH molar ratios used and the topologies obtained were ∞ (MIL-96), 9.1, 6.1 and 3.9 (mixture of MIL-100 and MIL-96), 3.4 (mixture of MIL-100 and MIL-110) and 1.5 (MIL-100); see Table 2. Closed symbols represent adsorption and open symbols desorption branch.

According to the N_2_ isotherms acquired at 77 K, the lower the amount of ethanol, the broader the pore size distribution in the obtained materials (see Fig. 6 and 7). Samples synthesized at pH 2.5 from mixtures of H_2_O and EtOH with molar ratios of 1.5 (pure MIL-100), 3.4 (mixture of MIL-100 and MIL-110) and 3.9 (mixture of MIL-100 and MIL-96) exhibited the narrowest BJH pore size distributions, which are centered at 33 Å (see Fig. 6).

**Fig. 8:**
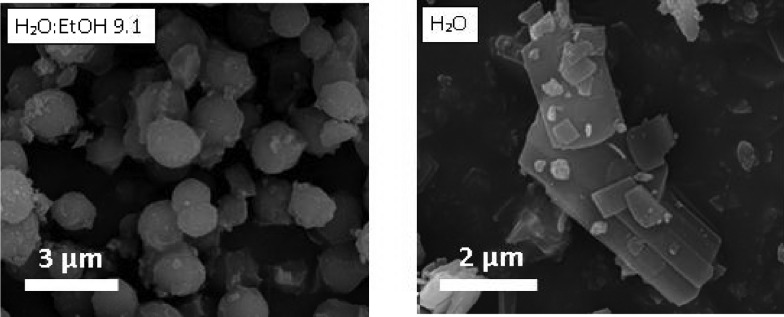
SEM images of the MIL-96 samples obtained at pH 2.5 in a mixture of H_2_O and EtOH with an H_2_O/EtOH molar ratio of 9.1 and in distilled water, both with a CTAB/Al molar ratio of 0.6.

When the synthesis was carried out in distilled water and no ethanol was added, the relative peak intensities of the XRD pattern were significantly different compared to those of the theoretically simulated XRD pattern of the MIL-96 topology. The XRD pattern of the sample obtained in water media (Fig. S10†) exhibits a strong increase in the relative intensity of the reflection associated with the (0 0 2) plane, which indicates the preferred crystal orientation of the (0 0 *l*) planes. To confirm this observation, powder pattern refinements were performed and the preferential orientation was modeled using the March-Dollase function. The powder XRD refinement results (see Table S2 and Fig. S11†) confirm a 0 0 1 preferential crystal orientation, the (0 0 1) plane running parallel to the bipyramidal cages of the MIL-96 topology (see Fig. S12†). Interestingly, the refined *G*-parameters of the March–Dollase function were equal to 0.51, indicating a lamellar particle morphology, in good agreement with the SEM micrographs of this sample and in clear contrast to the ill-defined hexagonal particles formed under “standard” synthesis conditions (see Fig. 8). In the case of MIL-96, the addition of surfactant did not affect the morphology of the MOF particles.

## Discussion

In this work, the synthesis of Al trimesate MOFs in the presence of the well-known cationic surfactant CTAB was studied to investigate the formation of MOFs with hierarchical porosity. Thus, in order to create mesoporosity, the concentration of surfactants used in all the experiments here presented is well above the critical micelle concentration (CMC) for CTAB in water ethanol mixtures.^49,50^ On the one hand, the configuration of the surfactant should have a clear effect on the kinetics of formation of the MOF; on the other hand, the concentration of co-solvents (ethanol in our case) and changes in pH have a strong effect on both MOF precursor solubility and micelle size and configuration.

We speculate that during the synthesis the ligand is incorporated in the hydrophobic interior region of the micelles leading to the expansion and deformation of the micelles formed. It has been shown that during the synthesis of mesoporous aluminas, an increase in the concentration of EtOH completely suppresses the formation of macropores.^50^ This effect is attributed to the fact that polar, protic cosolvents tend to decrease the aggregation and/or aggregate size of CTAB, leading to highly porous, disordered mesoporous materials.^49^ We therefore speculate that an increase in the concentration of EtOH has a twofold effect: (i) it reduces the size of the CTAB aggregates, leading to a faster exchange between the hydrophilic (Al-containing) phase and the hydrophobic (linker containing) phase as a result of the larger exchange area and (ii) it solubilizes the organic linker better. Because of the small aggregates and fast kinetics, very small MIL-100 particles are formed that lead to the creation of a secondary mesoporosity in the system. The smaller the MIL-100 particles, the better their packing and therefore the narrower the pore size distribution. Indeed, the samples here synthesized with high EtOH concentrations resulted in more homogeneous pore size distributions, while the samples synthesized under low EtOH concentrations present hardly any mesoporosity.

Furthermore, slight changes in pH will have the same effect: the higher the initial pH, the smaller the aggregates and the faster the ligand deprotonation, leading to the formation of hierarchical MOFs.

Finally, it is also important to stress that, at least in the case of Al based MOFs, it seems quite difficult to synthesize structures with a large degree of meso-order as SBA-15 or MCM-41 and non-ordered interparticle mesoporous voids were observed instead. In fact, several examples of hierarchical MOFs composed of nanoparticles have been published^28,51^ and just in a few cases long-ordered mesopores were achieved.^27,30^ In this sense, the interaction between template, co-solvent(s), metal cations and organic linker in solution deserves special attention and more efforts should be devoted to this interesting topic.

## Conclusions

Changes in the pH and in the H_2_O/EtOH molar ratio in the synthesis of Al trimesates in the presence of CTAB affect both micelle configuration and linker solubility, giving rise to 3 different topologies: MIL-96, MIL-100 and MIL-110. MIL-110 was obtained without additives to control the pH and at lower temperatures than those commonly reported. Furthermore, MIL-100 with particle sizes as small as 30 ± 10 nm was synthesized. In this latter case, the sample possesses hierarchical mesoporosity, with micropores being related to the MOF topology and mesopores related to interparticle voids. The size of CTAB aggregates in solution determines the degree of mesoporosity of the final sample and the aluminium trimesate phase formed: once the critical micelle formation concentration is reached, small aggregates formed at large CTAB/EtOH ratios resulting in the formation of MIL-100 composites assembled by the agglomeration of small MOF nanoparticles. Finally, platelets of MIL-96 were observed for the first time with a (001) preferential crystal orientation.

Our results demonstrate the importance of interaction at the molecular scale between template, co-solvents(s), metal cations and organic linker in solution. Understanding such interactions is crucial for the design of hierarchical MOFs.
