# Supplementary material for: Metal organic framework synthesis in the presence of surfactants: towards hierarchical MOFs?
Source: CrystEngComm. 2015 Jan 23;17(7):1693–700. doi: 10.1039/c4ce02324b (PMC4456781; doi:10.1039/c4ce02324b)
Supplement: Supplementary file 1 [file CE-017-C4CE02324B-s001.pdf]

Electronic Supplementary Material (ESI) for CrystEngComm.
This journal is © The Royal Society of Chemistry 2015

Supporting information

Metal Organic Framework synthesis in the presence of surfactants: towards hierarchical MOFs?

B. Seoane [*a], A. Dikhtiarenko [a], A. Mayoral [c], C. Tellez [b], J. Coronas [b], F. Kapteijn [a] and J. Gascon [*a]

[a] Catalysis Engineering, ChemE, Delft University of Technology, Julianalaan 136, 2628 BL Delft, The Netherlands.

[b] Chemical and Environmental Engineering Department and Nanoscience Institute of Aragon (INA), Universidad de Zaragoza, Mariano Esquillor, 50018, Edificio I+D, Zaragoza, Spain.

[c] Advanced Microscopy Laboratory (LMA), Nanoscience Institute of Aragon (INA), Universidad de Zaragoza, Mariano Esquillor, 50018, Edificio I+D, Zaragoza, Spain.

*Corresponding authors: B.SeoanedelaCuesta@tudelft.nl, j.gascon@tudelft.nl

Table. S1 Synthesis conditions for Al_BTC crystallization.

Run	H_2O/EtOH molar ratio	CTAB/Al molar ratio	T [°C]	Time [h]	pH	Base
Al_BTC 1	3.4	0.6	120	12	2.1	-
Al_BTC 2	3.4	0.6	120	12	2.2	TMAOH
Al_BTC 3	3.4	0.6	120	12	2.4	TMAOH
Al_BTC 4	3.4	0.6	120	12	2.5	TMAOH
Al_BTC 5	3.4	0.6	120	12	2.6	TMAOH
Al_BTC 6	3.4	0.6	120	12	2.7	TMAOH
Al_BTC 7	3.4	0	120	12	2.5	TMAOH
Al_BTC 8	∞	0.6	120	12	2.5	TMAOH
Al_BTC 9	9.1	0.6	120	12	2.5	TMAOH
Al_BTC 10	6.1	0.6	120	12	2.5	TMAOH
Al_BTC 11	3.9	0.6	120	12	2.5	TMAOH
Al_BTC 12	1.5	0.6	120	12	2.5	TMAOH
Al_BTC 13	9.1	0.3	120	12	2.5	TMAOH
Al_BTC 14	9.1	1.2	120	12	2.5	TMAOH

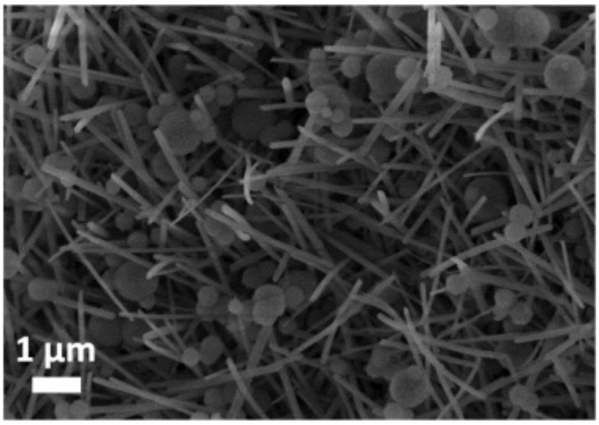

Fig. S1 SEM images of the MIL-110 sample obtained at pH 2.1 and 120°C after 12 h from a synthesis solution with CTAB/Al and H_2O/EtOH molar ratios of 0.6 and 3.4, respectively.

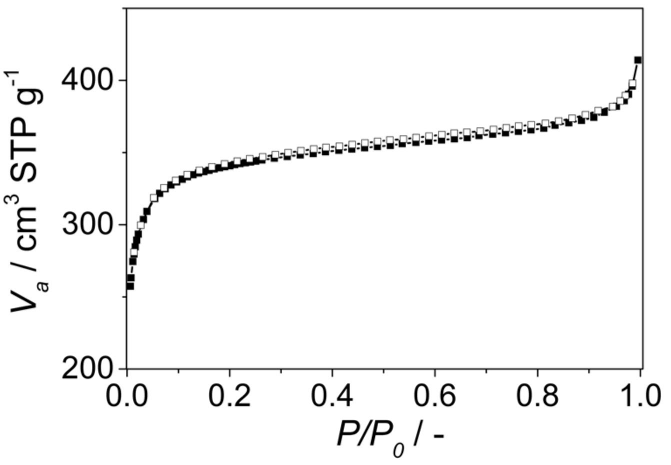

Fig. S2 N_2 adsorption isotherm acquired at 77 K for the MIL-110 sample obtained at pH 2.1 from a synthesis solution with CTAB/Al and H_2O/EtOH molar ratios of 0.6 and 3.4.

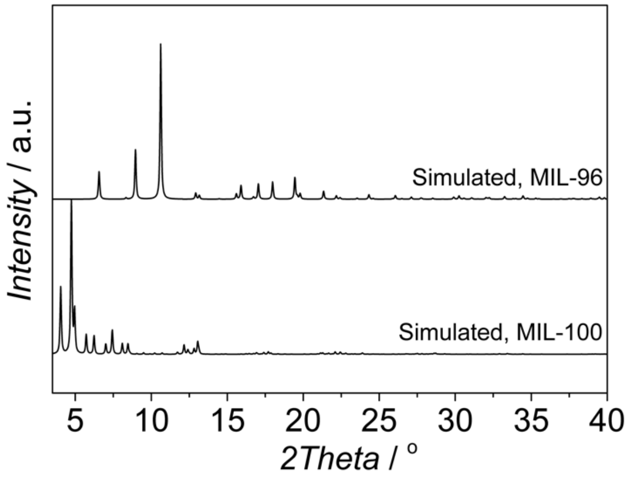

Fig. S3 Simulated patterns of MIL-96 and MIL-100 calculated from the data previously reported by Loiseau *et al.* [1] and Volkringer *et al.* [2]

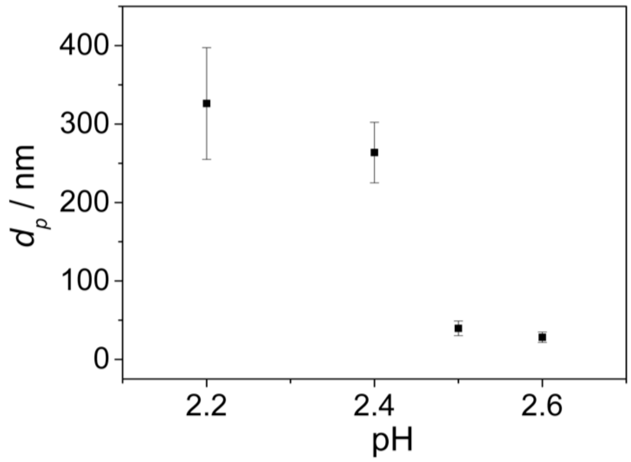

Fig. S4 Particle size of MIL-100 calculated from the SEM images (average of 100 particles) as a function of the pH.

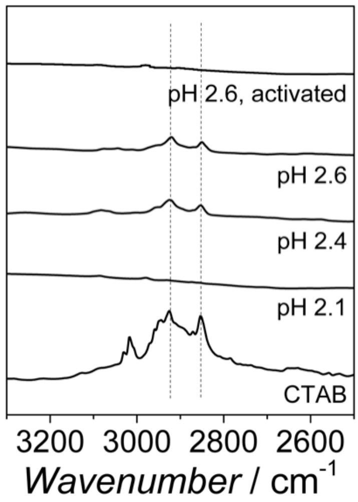

Fig. S5 FTIR of the samples obtained at different pH: 2.1 (MIL-110), 2.4 (MIL-100) and 2.6 (mixture of MIL-100 and MIL-96) from synthesis solutions with CTAB/Al and H_2O/EtOH molar ratios of 0.6 and 3.4, respectively. The term activated refer to the sample treated with EtOH under reflux and dried at 130 ^{o}C overnight.

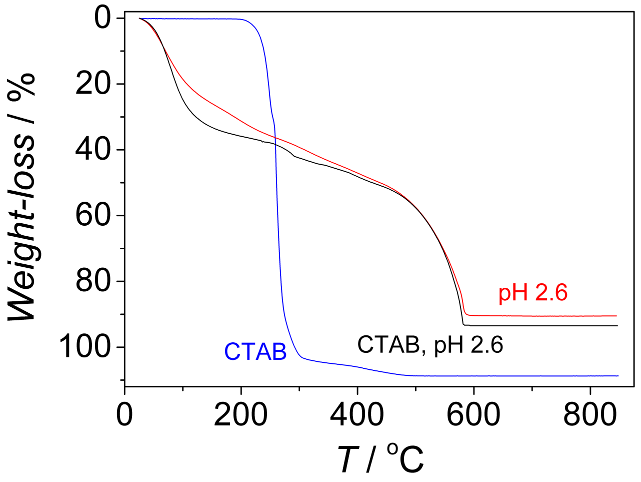

Fig. S6 TGA of the samples obtained at pH 2.6 (mixture of MIL-100 and MIL-96) from synthesis solutions with CTAB/Al molar ratios of 0 and 0.6 and H_2O/EtOH 3.4. The TGA of the cationic surfactant CTAB has also been included for comparison.

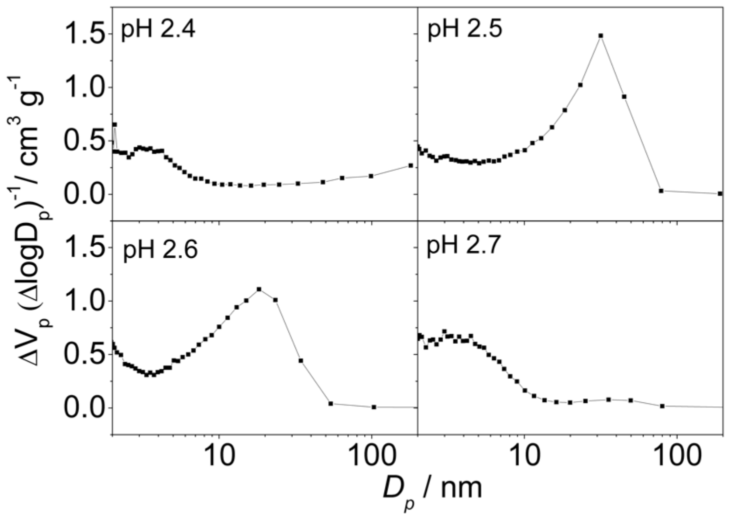

Fig. S7 BJH pore size distribution curves of the samples obtained at different pH from synthesis solutions with CTAB/Al and H_2O/EtOH molar ratios of 0.6 and 3.4, respectively. The pH values used and the topologies obtained were 2.1 (MIL-110), 2.2 (MIL-100), 2.4 (MIL-100), 2.5 (mixture of MIL-100 and MIL-110) and 2.6 and 2.7 (mixture of MIL-100 and MIL-96).

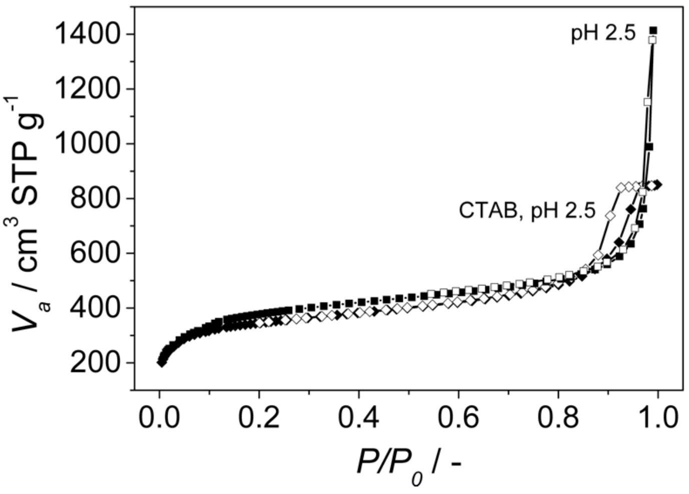

Fig. S8 N_2 adsorption isotherm @ 77 K of the samples obtained at pH 2.5 from a synthesis solution with an H_2O/EtOH molar ratio of 3.4 with and without CTAB.

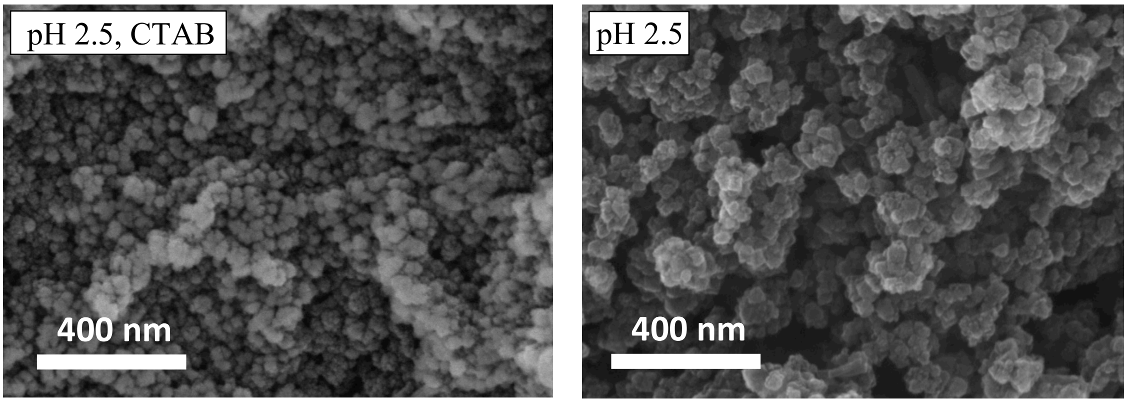

Fig. S9 SEM images of the samples obtained at pH 2.5 from a synthesis solution with an H_2O/EtOH molar ratio of 3.4 with and without CTAB.

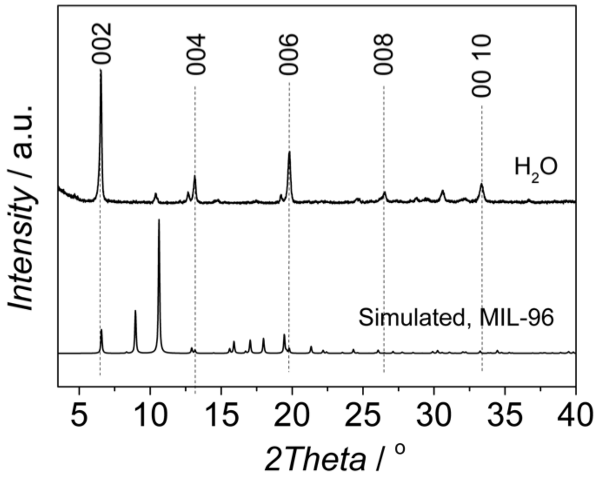

Fig. S10 XRD diffraction patterns of the sample synthesized in distilled water at pH 2.5 with CTAB/Al molar ratio of 3.4 together with the simulated MIL-96 pattern for comparison.

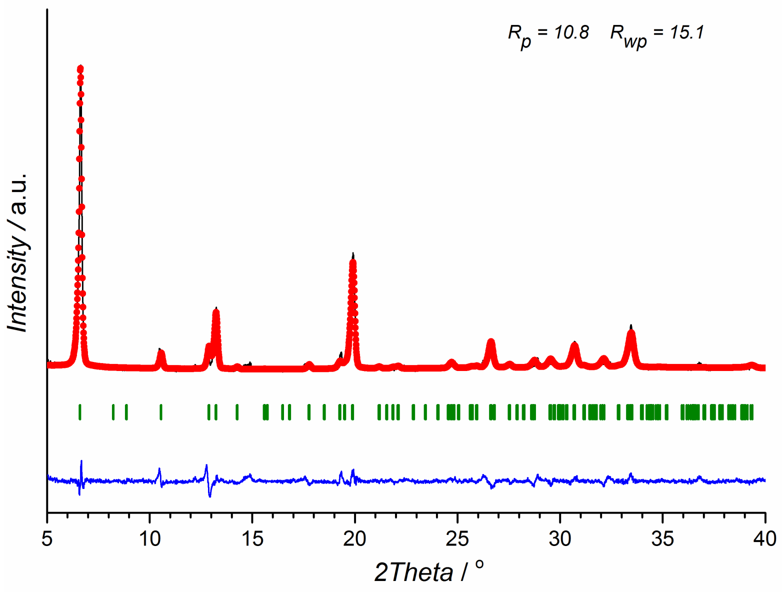

Fig. S11 Le Bail refinement plots for MIL-96(Al) obtained at pH 2.5 in water media. Experimental data, calculated data, and the difference between them are represented in black, red and blue, respectively. The Bragg positions of the peaks are represented as green sticks.

Table. S2 The unit cell and Le Bail refinement parameters of MIL-96(Al) obtained at pH 2.5 in distilled water.

Compound	MIL-96(Al)
Crystal System	Hexagonal
Space Group	*P63/mmc*
$a = b$ (Å)	14.439 (2)
c (Å)	31.130 (2)
$\alpha = \beta$ (°)	90
γ (°)	120
V (Å^3)	5459.3 (1)
$2\vartheta_{\text{interval}}$ (°)	5-40
$R_p{}^{i}$, $R_{wp}{}^{ii}$	0.108, 0.151
G_1	0.51

$^{i}R_P = \sum_i |y_{i,0} - y_{i,c}| / \sum_i |y_{i,0}|$; $^{ii}R_{wp} = \left[\sum_i w_i (y_{i,o} - y_{i,c})^2 / \sum_i w_i (y_{i,o})^2\right]^{1/2}$

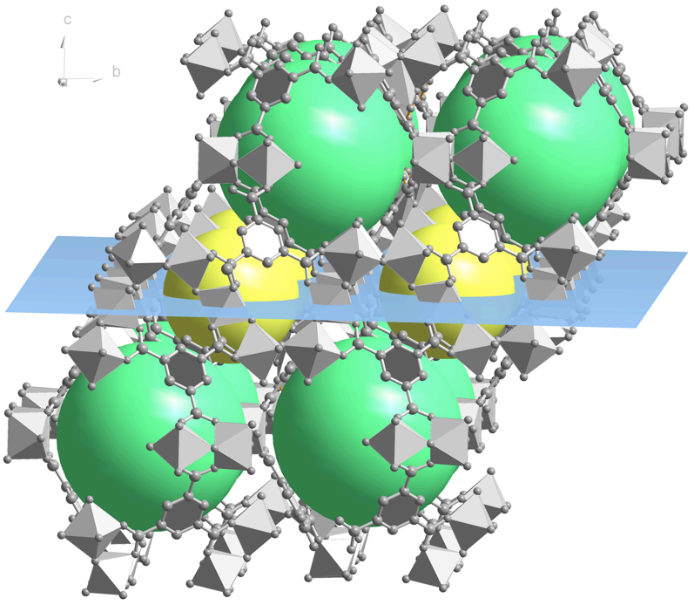

Fig. S12 Perspective drawing of the MIL-96(Al) topology with a (001) plane propagating through the structure. The colored spheres represent two types of cages: ≈ 420 Å^3 (yellow) and ≈ 635 Å^3 (green).

In order to assess the optimum amount of surfactant to obtain narrow pore size distributions, 3 different CTAB/Al^{3+} molar ratios were employed: 0.3, 0.6 and 1.2. The narrowest pore distributions were observed for the lowest CTAB/Al^{3+} ratio with pore size distributions centered at 18 nm (Fig. S.12 and Fig. S13). Moreover, when CTAB/Al^{3+} was decreased from 1.2 to 0.3, the S_{EXT}/S_{INT} ratio increased from 0.7 to 1.3, respectively. It seems that a decrease in the amount of CTAB in the synthesis solution led to a higher mesoporosity together with a narrower BJH pore size distribution.

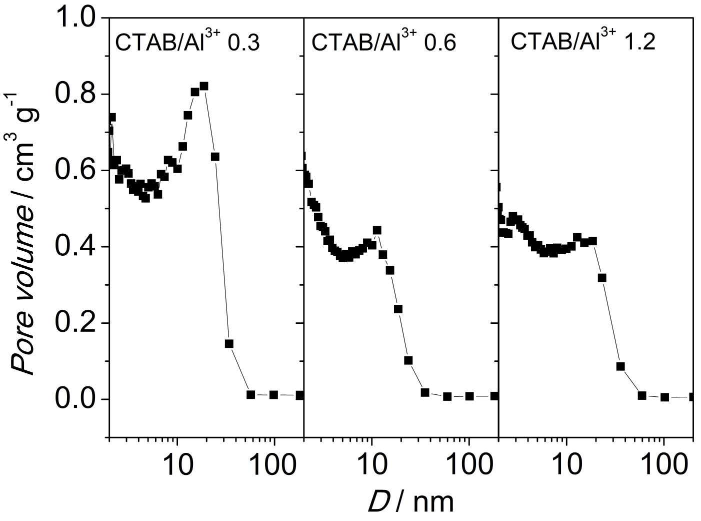

Fig.S13 BJH pore size distribution curves of the samples synthesized at pH 2.5 from synthesis solutions with H_2O/EtOH molar ratio of 9.1 and different amounts of CTAB.

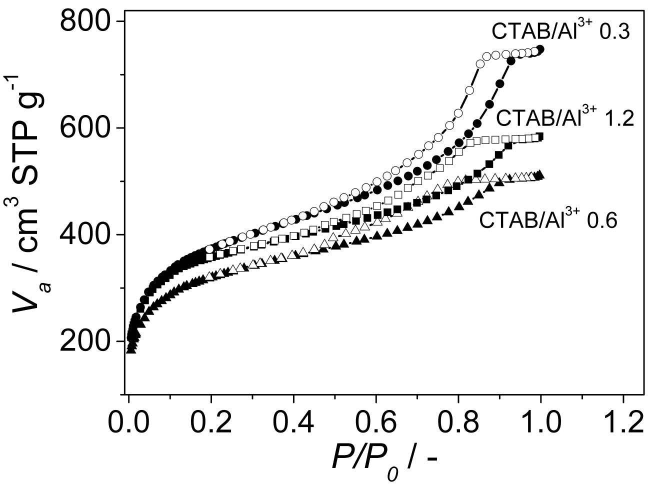

Fig. S14 N_2 adsorption isotherms measured at 77 K for the samples obtained at pH 2.5 from synthesis solutions with H_2O/EtOH molar ratio of 9.1 and different amounts of CTAB. Closed symbols represent adsorption and open symbols desorption branch.

[1] T. Loiseau, L. Lecroq, C. Volkringer, J. Marrot, G. Ferey, M. Haouas, F. Taulelle, S. Bourrelly, P.L. Llewellyn, M. Latroche, MIL-96, a porous aluminum trimesate 3D structure constructed from a hexagonal network of 18-membered rings and mu(3)-oxo-centered trinuclear units, Journal of the American Chemical Society, 128 (2006) 10223-10230.

[2] C. Volkringer, D. Popov, T. Loiseau, G. Ferey, M. Burghammer, C. Riekel, M. Haouas, F. Taulclle, Synthesis, Single-Crystal X-ray Microdiffraction, and NMR Characterizations of the Giant Pore Metal-Organic Framework Aluminum Trimesate MIL-100, Chemistry of Materials, 21 (2009) 5695-5697.
